# The Effect of Coconut and Frankincense Oils on the Biofilm Growth of *Streptococcus mutans*

**DOI:** 10.12688/f1000research.168621.1

**Published:** 2025-09-01

**Authors:** Nada Alghamdi, Lama Alshamrani, Safa Alboryah, Jomana Fayez Alsenan, Turki Alshehri, Naif N. Abogazalah, Abdulrahman A. Balhaddad

**Affiliations:** 1 College of Dentistry, Imam Abdulrahman Bin Faisal University, Dammam, 31441, Saudi Arabia; 2 Department of Clinical Sciences, College of Dentistry, Ajman University, Ajman, United Arab Emirates; 3 Department of Substitutive Dental Sciences, College of Dentistry, Imam Abdulrahman Bin Faisal University, Dammam, 31441, Saudi Arabia; 4 Department of Restorative Dental Sciences, College of Dentistry, King Faisal University, Al Ahsa, 31982, Saudi Arabia; 5 Department of Restorative Dental Sciences, College of Dentistry, Imam Abdulrahman Bin Faisal University, Dammam, 31441, Saudi Arabia

**Keywords:** Essential oils; biofilm; caries; gingivitis; mouthrinse.

## Abstract

**Background:**

This study aimed to investigate the antibiofilm effect of coconut and frankincense oils.

**Methods:**

Different types of coconut (organic refined, cocos nucifera, organic virgin, and organic extra virgin) and frankincense (frankincense pure essential oil in fractionated coconut oil and uplifting frankincense pure essential) oils were investigated. Serial dilutions (1:3, 1:6, 1:12, 1:24, 1:48, 1:96, and 1:192) were created from each oil and incubated with an overnight culture of *S. mutans.* The total growth and biofilms absorbance were measured at 595 and 490 nm, respectively. One-way ANOVA and Tukey tests were used for data analysis.

**Results:**

The greatest biofilm inhibition was observed in the uplifting frankincense pure essential oil at 1:3 dilution (0.67±0.12), which was significantly lower ( *p*<0.01) than the control (1.51 ± 0.07). In addition, organic refined (0.96±0.13), organic virgin (1.21±0.28), and organic extra virgin (1.07±0.17) were associated with less biofilms compared to the control, but without a statistical significance. Frankincense pure essential oil in fractionated coconut oil and cocos nucifera coconut oil did not show biofilm inhibition.

**Conclusions:**

Organic refined, organic virgin, and extra virgin coconut oils, and uplifting frankincense essential oil, effectively reduced *S. mutans* levels *in vitro*, with the highest amount of biofilm reduction associated with uplifting frankincense essential oil.

## 1. Introduction

Dental caries is a disease influenced by multiple factors. Elements such as diet, the shape and structure of teeth, the oral environment, and the presence of certain microorganisms all contribute to the development of dental caries. ^1, 2^ Among such microorganisms, *Streptococcus mutans* ( *S. mutans*) is a facultative anaerobic, gram-positive bacterium known for its strong association with dental caries due to its high virulence. ^3^ Its ability to adhere to tooth enamel, produce acidic byproducts, store glycogen, and generate extracellular polysaccharides contributes significantly to its role in tooth decay. ^4^ Several methods have been investigated to prevent dental caries by targeting the formation of cariogenic biofilms through the application of antimicrobial agents. ^5^

Mechanical tooth cleaning is the most widely accepted and dependable approach for maintaining oral hygiene. In addition to mechanical tooth cleaning, research has concentrated on chemotherapeutic agents aimed at reducing or preventing dental caries. ^6, 7^ With the growing concern regarding the emergence of pathogens with antimicrobial resistance in dentistry and medicine, several investigations have explored the capabilities of natural products in preventing biofilm-triggered diseases. ^8, 9^ Oil pulling is a traditional oral hygiene practice, with coconut oil ( *cocos nucifera*) being one of the most frequently used oils for this method. ^10^ Oil pulling, traditionally referred to as ‘Kavalagraha’ or ‘Kavala Gandoosha’ in the ancient Ayurvedic text *Charaka Samhita*, is a practice that involves swishing natural oils in the mouth. This technique is believed to help eliminate harmful bacteria, fungi, and other microorganisms from the teeth, gums, throat, and oral cavity. ^11^

Coconut oil is rich in medium-chain saturated fatty acids, which compared to long-chain fatty acids, are more readily absorbed and metabolized by the body. This is due to their smaller size, greater solubility, and enhanced resistance to oxidation. ^10, 12^ Coconut oil is composed of about 92% saturated fats, with lauric acid making up nearly half of that content. Lauric acid is known for its antibacterial and antifungal properties. ^10, 13^ Research has demonstrated that coconut oil exhibits strong antimicrobial effects against various microorganisms. ^14^ Studies evidenced that coconut oil is also effective against *S. mutans* and *C. albicans* in an *in vitro* oral biofilm model. ^14, 15^

Frankincense oil, derived from *Boswellia* species, is another pulling oil that has shown a long-standing historical and medicinal significance, notably in traditional medicine, due to its aromatic properties and therapeutic potential. ^16^ Its active component, boswellic acids like acetyl-11-keto-β-boswellic acid (AKBA), plays a significant role in supporting oral and dental health by exhibiting antimicrobial and anti-inflammatory properties. ^17^ Research shows that frankincense, especially from Boswellia serrata, effectively inhibits key oral pathogens such as *Porphyromonas gingivalis* and *Enterococcus faecalis*, which are involved in periodontitis. ^17^

Coconut oil and frankincense oil pulling has been reported to help reduce plaque-related gingivitis and bad breath. ^18^ However, there is limited scientific evidence regarding their impact on *S. mutans*, the bacteria primarily involved in the onset and development of dental caries. Besides, with the presence of different forms of these two oils, investigate the most anticariogenic form worths to be investigated. Therefore, the current study aimed to evaluate the effect of different types of coconut and frankincense oils against *S. mutans* biofilm growth. Besides, this study investigates the synergetic effect of the two oils against *S. mutans* biofilms. We hypothesized that coconut and frankincense oils, and their combination would significantly reduce the *S. mutans* biofilm growth.

## 2. Materials and methods

### 2.1 Study design and sample size calculation

In this study, four different types of organic coconut oil were used: (A) organic refined coconut oil, (B) fractionated liquid coconut oil (cocos nucifera), (C) organic virgin coconut oil, (D) organic extra virgin coconut oil. Also, two types of frankincense oil were used: (E) frankincense pure essential oil in fractionated coconut oil, (F) uplifting frankincense pure essential oil. The details of the selected oils and their manufacturers’ information are found in Table 1. Prior studies ^19– 21^ indicated that the standard deviation for absorbance measurements associated with biofilm formation is roughly 0.15. As a result, this study was structured to attain an 80% power level for detecting a significant difference at a 5% significance threshold. To achieve this, three separate experiments were conducted, each involving 3 to 4 samples, culminating in a total of 9 to 12 samples per group.

**Table 1.** List of coconut and frankincense oils used in this study.

MANIFUCTURER	COUNTRY	PRODUCT NAME	CONTENT	OIL ORIGIN
Packaged for NOW Foods	Canada	Liquid coconut oil	Cocos Nucifera (Coconut) oil	Malaysia
Distributed by Marico Middle East FZE, Dubai, UAE	Sri Lanka	Virgin coconut iol	100% organic virgin coconut oil	Sri Lanka
Distributed by LA Tourangelle, INC. California	USA	Organic refined coconut oil	Organic refined coconut oil	USA
Distributed by Nature’s Way Brands, LLC Green Bay, WI 54311 USA	philippines	Organic extra virgin	Organic extra virgin coconut oil contains tree nuts	philippines
Distributed by Nature’s Truth LLC Bohemia, USA	USA	Uplifting Frankincense Pure Essential oil	Pure rankincense oil (Boswellia serrata)	USA
Distributed by Frontier co-op po, Norway, USA	USA	Frankincense Pure essential oil in fractionated coconut oil	Caprylic/Capric triglyceride (fraction of coconut oil) 4% Boswellia sacra (Frankincense) oil	USA

### 2.2 Preparation of the oils’ dilutions

Each type of oil was mixed with brain-heart infusion (BHI) broth supplemented with 2% sucrose at the following dilutions: 1:3, 1:6, 1:12, 1:24, 1:48, 1:96, 1:192. The mixtures were used immediately after mixing. The experimental design included six main experimental groups according to the type of oil then another seven subgroups according to the dilution. A group consisting of BHI supplemented with 2% sucrose only was used as a control.

### 2.3 The impact of oils on *S. mutans* biofilms

An overnight culture of *S. mutans* was prepared in BHI broth. The optical density (OD) of the culture was adjusted to 0.9 to standardize the bacterial concentration. All experimental and control samples were incubated with an overnight culture of *S. mutans* for 24 hours at a ratio of 20:1 (broth to bacterial culture). Subsequently, 10 μL of the adjusted *S. mutans* culture was added to 190 μL of either different type of oil dilution (oil mixed with BHI broth supplemented with 2% sucrose) at 1:3, 1:6, 1:12, 1:24, 1:48, 1:96, and 1:192 dilutions or the control group (BHI with 2% sucrose only) inside the wells of a 96-well plate. A sterility control of only BHI with 2% sucrose was also applied to detect any possible contamination. The 96-well plates were then incubated aerobically at 5% CO _2_ for 24 hours. After incubation, the total absorbance (planktonic and biofilm) was measured at 595 nm using a spectrophotometer (SpectraMax M5, Molecular Devices, Sunnyvale, CA, USA). ^19– 21^

Following this, the planktonic cells were removed, and the remaining adherent biofilm was treated with 200 μL of 10% formaldehyde for 30 minutes. The wells were then rinsed three times with deionized water to eliminate residual formaldehyde. To visualize the biofilm, 200 μL of 0.5% crystal violet solution was added and left to stain for 30 minutes. Afterward, excess dye was removed through three additional washes, leaving only the stained biofilm. And the spectrophotometer measurement at 490 nm was used to estimate the biofilm absorbance. ^19– 21^

### 2.4 Statistical analysis

Data were expressed as means ± standard deviations, based on at least three biological replicates to provide descriptive statistics. The Shapiro-Wilk test was employed to assess the normality of the data distribution. To compare the effects of coconut and frankincense oils on the biofilm formation and total growth of *S. mutans*, one-way ANOVA followed by Tukey’s post hoc tests were utilized (using Sigma Plot 12.0; SYSTAT). A p-value of less than 0.05 was deemed statistically significant.

## 3. Results

### 3.1 Effect of different types of coconut and frankincense oils on *S. mutans* total growth

**Figure 1.**
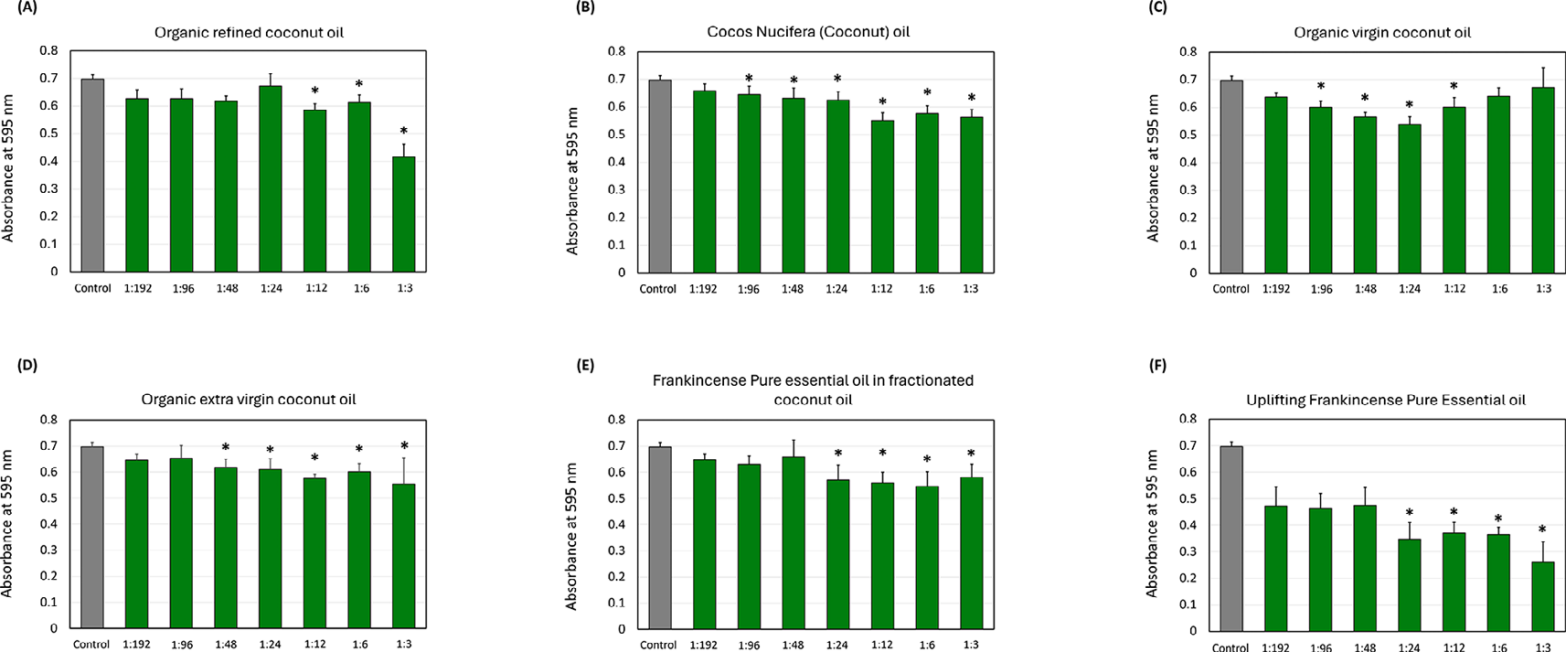
Effect of the coconut and frankincense oils on *Streptococcus mutans* total growth. The impact of (A) organic refined coconut oil (B) cocos nucifera, (C) organic virgin coconut oil, (D) organic extra virgin coconut oil, (E) frankincense pure essential oil in fractionated coconut oil, and (F) uplifting frankincense pure essential oil on *S. mutans* total growth. Each group consisted of 3 wells, and the experiment was repeated three times (n = 9). Asterisks indicate a significant difference compared to the control samples with no treatment.

Figure 1 illustrates the effect of coconut and frankincense oils on total growth. Organic refined coconut oil ( Figure 1A) showed the absorbance values decrease with increasing concentrations, showing significant reduction ( *p *< 0.05) at the 1:12, 1:6, and 1:3 dilutions compared to the control. Similar downward trends were observed for cocos nucifera oil ( Figure 1B), with significant reductions at all the dilutions except for the 1:192 dilution. For organic virgin coconut oil ( Figure 1C), absorbance levels decline with increasing concentration, with significant reduction ( *p* < 0.05) starting from the 1:96 to 1:12 dilutions when compared to the control, but no significant change in the absorbance at 1:6, and 1:3 dilutions. In addition, organic extra virgin coconut oil ( Figure 1D), particularly at the 1:48, 1:24, 1:12, 1:6, and 1:3 dilutions, showed significant reduction ( *p* < 0.05) in the total growth compared to the control.

Frankincense pure essential oil in fractionated coconut oil ( Figure 1E) showed a marked decrease in the OD across all concentrations, with the significant reduction at 1:24, 1:12, 1:6, and 1:3. Similarly, the uplifting frankincense pure essential oil ( Figure 1F) demonstrated the most amount of total growth reduction, which was observed with increased concentration, especially at the 1:24, 1:12, 1:6, and 1:3 dilutions ( *p* < 0.05).

### 3.2 Effect of different types of coconut oil on biofilm only *S. mutans*

**Figure 2.**
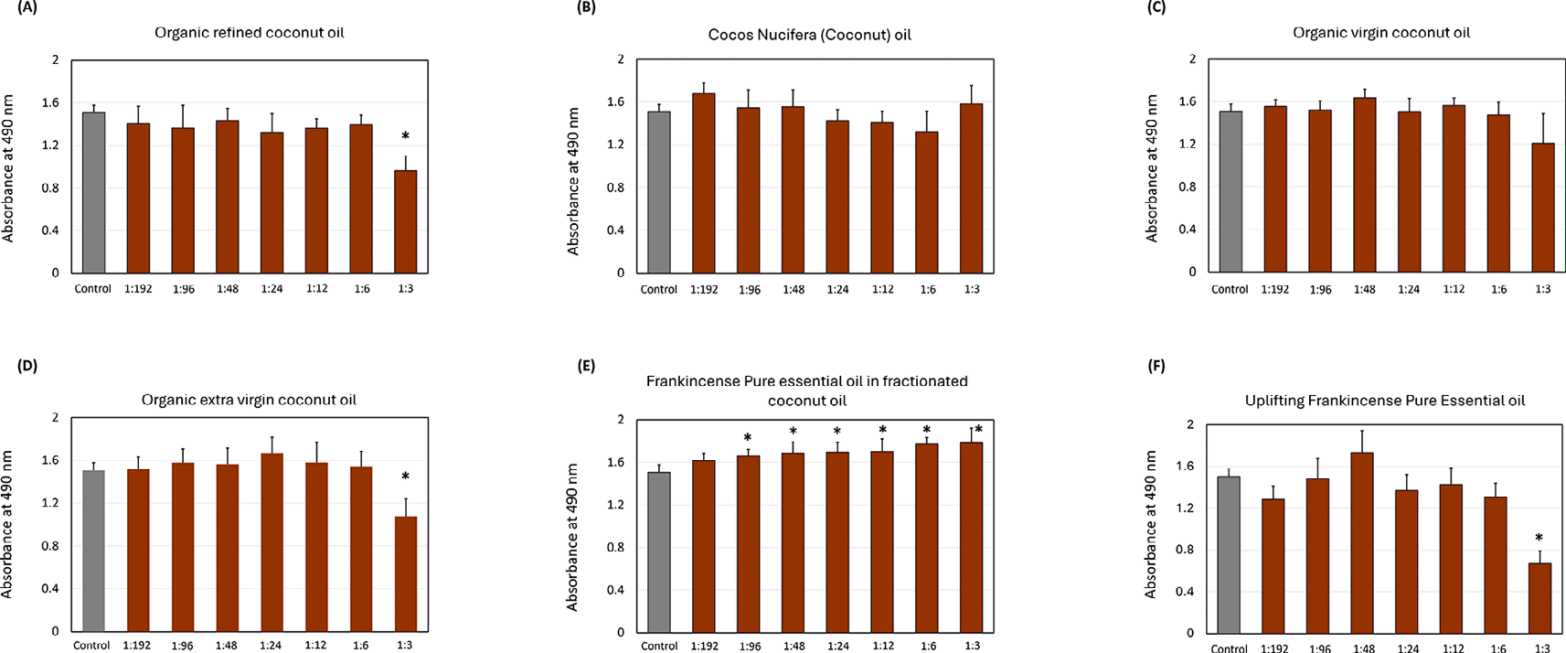
Effect of the coconut and frankincense oils on *Streptococcus mutans* biofilms. The impact of (A) organic refined coconut oil (B) cocos nucifera, (C) organic virgin coconut oil, (D) organic extra virgin coconut oil, (E) frankincense pure essential oil in fractionated coconut oil, and (F) uplifting frankincense pure essential oil on *S. mutans* biofilm formation. Each group consisted of 3 wells, and the experiment was repeated three times (n = 9). Asterisks indicate a significant difference compared to the control samples with no treatment.

Figure 2 illustrates the impact of oils on the biofilm growth of *S. mutans.* The organic refined coconut oil ( Figure 2A) led to a decrease in biofilm absorbance as the concentration increased, with the only significant reductions observed at the 1:3 dilution ( *p* < 0.05). Cocos nucifera and organic virgin coconut oils ( Figure 2B & 2C) exhibited a progressive decline in OD with higher concentrations, but the amount of reduction was not significant at any dilution. The organic extra virgin coconut oil ( Figure 2D) showed only significant reduction ( *p* < 0.05) at the 1:3 dilution.

Among the frankincense oils, pure frankincense essential oil in fractionated coconut oil ( Figure 2E) significantly increased the biofilm growth across most tested concentrations, with the strongest effects at 1:6, 1:12, and 1:3. Opposingly, the uplifting frankincense essential oil ( Figure 2F) reduced the biofilm growth at all the dilutions except at the 1:48 dilution, with the only significant biofilm, inhibition at the 1:3 dilution ( *p* < 0.05).

### 3.3 Comparison between different types of coconut oil and frankincense oil on biofilm only *S. mutans* at 1:3 dilution

**Figure 3.**
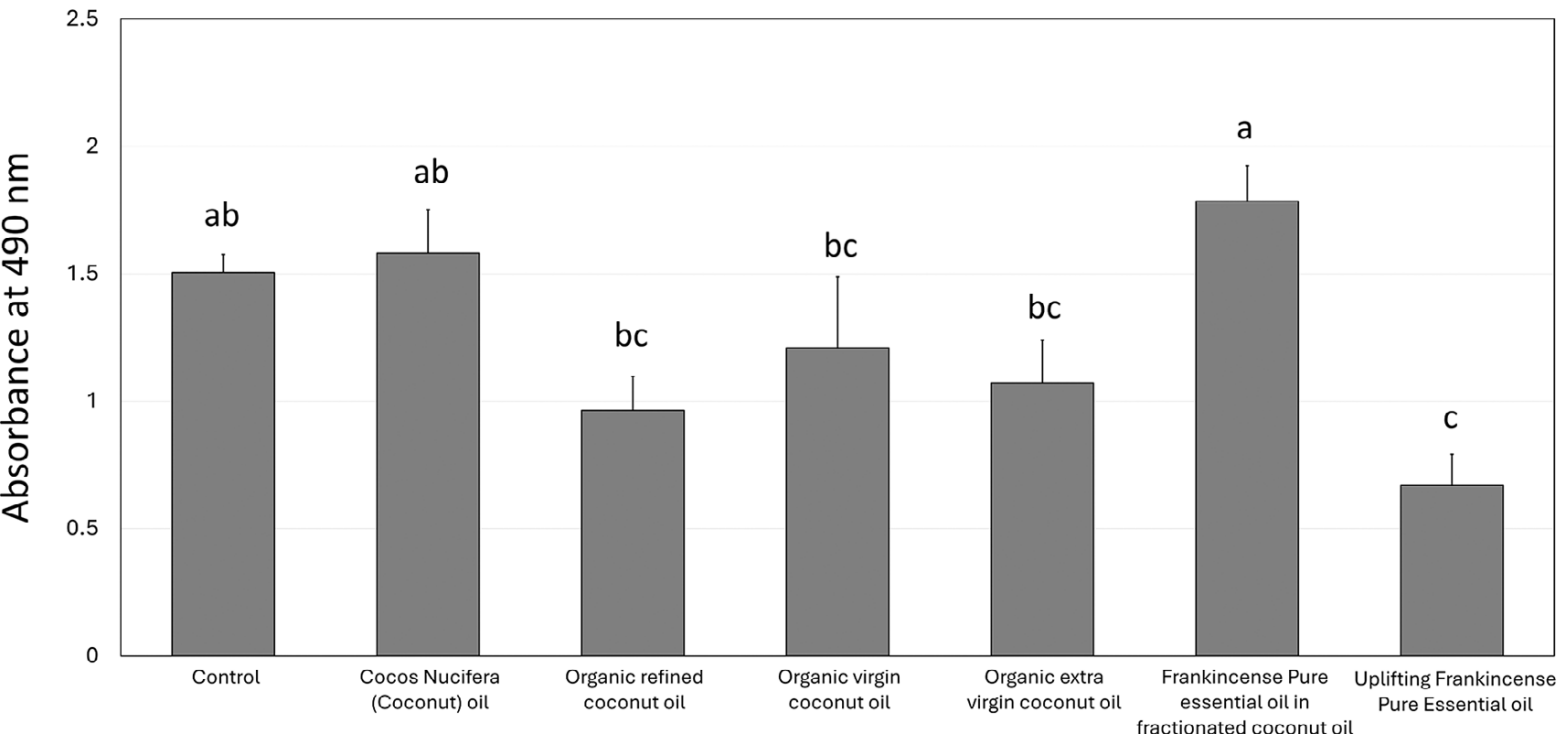
Effect of different types of coconut and frankincense oils on *Streptococcus mutans* biofilms at the 1:3 dilution. Each group consisted of 3 wells, and the experiment was repeated three times (n = 9). Different letters indicate significant differences between the oils.

Figure 3 illustrates the effects of various oils on *S. mutans* levels at the 1:3 dilution. The highest reduction observed in the group was treated with uplifting frankincense pure essential oil, which had an absorbance of 0.67 nm ± 0.12 nm, the lowest among all groups, and it was significantly lower ( *p* < 0.01) compared to the control (1.51 nm ± 0.07 nm). In addition, organic refined (0.96 nm ± 0.13 nm), organic virgin (1.21 nm ± 0.28 nm), and organic extra virgin (1.07 nm ± 0.17 nm) were associated with less biofilms compared to the control, but without a statistical significance. The group treated with frankincense pure essential oil in fractionated coconut oil and cocos nucifera coconut oil were associated with anincrease in the biofilm growth compared to the control.

## 4. Discussion

This study evaluated the antimicrobial effects of different coconut and frankincense oils preparations on *S. mutans* in both planktonic and biofilm forms. Notably, organic virgin coconut, extra virgin coconut, organic refined coconut, and uplifting frankincense essential oils showed the strongest inhibitory effects, particularly at higher concentrations. While the other oils were associated with no reduction against the *S. mutans* biofilms. Therefore, the hypothesis was partially accepted. The findings of this study highlight the potential of natural oils as alternative or adjunctive agents in oral health care, especially in reducing cariogenic bacteria.

Given the rising interest in natural products and growing concerns about antimicrobial resistance, the use of compounds such as coconut oil and frankincense oil presents a promising alternative to conventional oral antiseptics. Alcohol-containing mouthwashes, while effective, are associated with drawbacks such as mucosal irritation, altered taste, and patient discomfort, particularly with long-term use. ^22, 23^ Furthermore, widespread reliance on synthetic agents like chlorhexidine has raised concerns about the potential for promoting antimicrobial resistance. ^24^ In contrast, natural oils have not been linked to resistance development and may offer a safer profile. ^25^ They may also preserve the balance of the oral microbiome more effectively than alcohol-based products. ^26^ Their multifunctional properties, including anti-cariogenic effects, low toxicity, and potential for long-term use, are valuable tools in preventive dentistry, particularly for individuals seeking holistic and gentler alternatives. ^27– 29^

Dental biofilms are the pathogenic form for oral microbes when causing dental caries or other biofilm-induced diseases. ^30^ Opposite to their planktonic form, bacteria embedded within the biofilms are 1,000 resistant to therapeutic agents and bioactive compounds, which can complicate controlling the biofilm and reducing its pathogenicity. ^31, 32^ Therefore, we intended in this study to investigate the effect of oils on *S. mutans* planktonic and biofilms forms to explore the potential of these oils against more different forms of oral microbes. Our findings support and expand upon existing evidence regarding the antimicrobial properties of coconut oil against *S. mutans.* Previous studies have attributed these effects to the high concentration of medium-chain fatty acids—particularly lauric acid—which disrupt bacterial cell membranes, leading to cell lysis and reduced viability. ^10^ Moreover, our observation that organic extra virgin coconut oil showed stronger biofilm reduction than other coconut oil types adds to findings by Alyami et al., ^33^ who emphasized medium-chain fatty acids ability to disrupt *S. mutans* within biofilms. This suggests that oil purity and extraction methods may influence antimicrobial efficacy, an area that has received limited attention in prior studies. By comparing different forms of coconut oil, our study refines current understanding and highlights the importance of oil quality in optimizing natural oral health interventions.

In contrast to coconut oil, frankincense oil is less extensively studied for its antimicrobial effects against *S. mutans*, though initial evidence suggests potential activity. Previous studies have highlighted frankincense’s anti-inflammatory and biofilm-disrupting properties, but direct evidence targeting *S. mutans* remains limited. ^34, 35^ Our findings help fill this gap by demonstrating that uplifting frankincense essential oil significantly reduced *S. mutans* biofilm levels, indicating promising antibacterial potential. However, the unexpected increase in OD observed with the pure frankincense essential oil in fractionated coconut oil suggests that formulation and carrier oils may influence efficacy. This highlights the complexity of essential oil interactions and the need for standardized preparations in oral applications. Compared to coconut oil, which has a well-established inhibitory effect on *S. mutans* in both planktonic and biofilm forms, ^10^ frankincense oil shows emerging but less consistent results. Thus, while our data supports its potential, further investigation is needed to clarify its mechanisms, optimal concentrations, and formulation for oral health use.

The results of this study have potential applications in the creation of oral care products that include coconut and frankincense oils. Given their demonstrated antimicrobial activity against *S. mutans*, these natural oils could be formulated into mouthwashes, toothpastes, or oil-based rinses aimed at caries prevention. Essential oils are already used in commercial dental products due to their broad-spectrum antimicrobial effects and lower side effect profiles compared to synthetic agents like chlorhexidine. ^36^ Some mouthwashes contain a combination of essential oils, including eucalyptus, tea tree, and clove oils, which have been shown to be effective against plaque and gingivitis. ^37^ Moreover, their inclusion may appeal to patients seeking alcohol-free and holistic oral hygiene solutions, especially those with sensitivities to conventional formulations. To ensure clinical efficacy, future product development should focus on optimizing delivery systems and conducting in *vivo studies.*

This study has some limitations that should be considered when interpreting the results. First, the experiment was conducted *in vitro*, which may not fully replicate the complex conditions of the oral cavity, including salivary flow, oral microbiome diversity, and host immune responses. Second, the study focused solely on *S. mutans*, while other cariogenic and commensal microorganisms play significant roles in oral biofilm ecology and caries development. Third, although different types and concentrations of coconut and frankincense oils were tested, variability in oil composition due to sourcing, processing, and brand differences could affect reproducibility and generalizability. Additionally, the mechanism of action was not directly investigated, limiting insights into how the oils exert their antibacterial effects. Future studies incorporating multi-species biofilm models, and advanced imaging or molecular techniques would strengthen the evidence for the therapeutic potential of these natural oils.

This study offered a comparative analysis of different types of coconut and frankincense oils against *S. mutans*, particularly in its biofilm form, which is clinically relevant for caries development. By highlighting the superior performance of organic extra virgin coconut oil and uplifting frankincense essential oil, the study identifies promising natural alternatives to conventional antimicrobials in oral care. Future research should build upon these findings through *in vivo* studies and clinical trials to assess real-world efficacy, safety, and patient compliance. Additionally, mechanistic studies exploring the specific bioactive compounds responsible for antimicrobial activity, such as lauric acid in coconut oil and boswellic acids in frankincense, would offer deeper insights. Expanding investigations to include multi-species biofilms and interactions within the oral microbiome could also better simulate clinical conditions. Finally, formulating standardized, stable delivery systems (e.g., mouthwashes, gels, or varnishes) will be critical for translating these natural products into practical oral health solutions.

## 5. Conclusion

Certain coconut and frankincense oils, particularly organic refined, organic virgin, and extra virgin coconut oils, and uplifting frankincense essential oil, effectively reduced *S. mutans* biofilms *in vitro.* The highest amount of biofilm reduction was associated with uplifting frankincense essential oil, which reduced the biofilm growth by 2.5-fold. These findings support their potential use as natural agents for caries prevention and oral health care. Opposingly, cocos nucifera coconut oil and frankincense pure essential oil did not induce antibiofilm effects against *S. mutans* biofilms.

## Author contributions

N.A., L.A., and S.A.: contributed to design, resources, acquisition and analysis, data curation, and drafted the manuscript, and critically revised the manuscript. J.F.A., T.A., N.N.A., and A.A.B.: contributed to the concept and design, acquisition, supervision, drafted the manuscript, and critically revised the manuscript. All authors have read and agreed to the published version of the manuscript.

## Institutional review board statement

Not applicable.

## Informed consent statement

Not applicable.

## Data Availability

Figshare. Coconut oil.xlsx https://doi.org/10.6084/m9.figshare.29875172.v2 ^38^ This project contains the following underlying data:
• An excel sheet that contains the full data for analysis Data are available under the terms of the Creative Commons Attribution 4.0 International license (CC-BY 4.0).
