# Supplementary material for: The Effect of Coconut and Frankincense Oils on the Biofilm Growth of Streptococcus mutans
Source: F1000Res. 2025 Sep 1;14:846. [Version 1] doi: 10.12688/f1000research.168621.1 (PMC12759288; doi:10.12688/f1000research.168621.1)
Supplement: Peer review history

# Reviewer report: Reviewer response for version 1

**DOI:** 10.5256/f1000research.185811.r439638

**Published:** 2026-01-02

**Authors:** Nurdiana Dewi

**Affiliations:** 1 Universitas Lambung Mangkurat, Banjarmasin, Indonesia

1. The research structure and main data are relevant and supported by appropriate references, and the results demonstrate new findings regarding effects on *Streptococcus mutans* biofilm formation

 2. As the suggestions:

- The authors may wish to ensure that the reporting of sample size is consistent between the methods and results sections, as the methods describe 9–12 samples per group, whereas the results report only 9 samples per group.
- The authors may also wish to temper the statement in the discussion regarding the potential of coconut oil and frankincense oil as alternatives to conventional oral antiseptics, as the conclusions are based on experiments using a single bacterial species ( *Streptococcus mutans*).

Is the work clearly and accurately presented and does it cite the current literature?

Yes

If applicable, is the statistical analysis and its interpretation appropriate?

Yes

Are all the source data underlying the results available to ensure full reproducibility?

Yes

Is the study design appropriate and is the work technically sound?

Yes

Are the conclusions drawn adequately supported by the results?

Yes

Are sufficient details of methods and analysis provided to allow replication by others?

Yes

Reviewer Expertise:

Microbiology; Pediatric Dentistry

I confirm that I have read this submission and believe that I have an appropriate level of expertise to confirm that it is of an acceptable scientific standard.

# Reviewer report: Reviewer response for version 1

**DOI:** 10.5256/f1000research.185811.r439644

**Published:** 2025-12-29

**Authors:** Morita Sari

**Affiliations:** 1 Universitas Muhammadiyah Surakarta, Surakarta, Central Java, Indonesia

**Comment (≈50 words):**

 The study demonstrates acceptable reproducibility through clearly defined materials and treatment protocols. The experimental design is appropriate for evaluating biofilm inhibition, using relevant controls and comparative groups. Data presentation appears systematic and measurable; however, greater detail on sample size, replication frequency, and statistical analysis would further strengthen reliability and reproducibility.

Is the work clearly and accurately presented and does it cite the current literature?

Partly

If applicable, is the statistical analysis and its interpretation appropriate?

Yes

Are all the source data underlying the results available to ensure full reproducibility?

Yes

Is the study design appropriate and is the work technically sound?

Yes

Are the conclusions drawn adequately supported by the results?

Yes

Are sufficient details of methods and analysis provided to allow replication by others?

Yes

Reviewer Expertise:

cariology, ecological caries, dental public health

I confirm that I have read this submission and believe that I have an appropriate level of expertise to confirm that it is of an acceptable scientific standard.

# Reviewer report: Reviewer response for version 1

**DOI:** 10.5256/f1000research.185811.r415947

**Published:** 2025-10-15

**Authors:** Marco Antonio Sánchez-Tito

**Affiliations:** 1 Universidad Peruana Cayetano Heredia, Lima District, Lima Region, Peru

Introduction: 

 Although *Streptococcus mutans* is a key microorganism in the development of dental caries, the manuscript should better reflect the complexity of the cariogenic process, emphasizing the diversity of bacterial species involved in the formation and maturation of dental biofilm, as well as the crucial role of *quorum sensing* mechanisms in regulating virulence and interspecies communication. Including several microbiological and molecular methods (e.g., culture-based assays, molecular identification, or biofilm quantification) with appropriate references would strengthen the methodological framework and demonstrate awareness of the multifactorial nature of caries pathogenesis.

 Material and Methods:

 In the Materials and Methods section, the authors should clearly specify the statistical software or computational method used for sample stimation. Additionally, the rationale for using only a negative control should be justified (why was a positive control not included?) The description of the bacterial culture conditions should also be corrected: the text should indicate that cultures were incubated anaerobically, not *aerobically* (likely a typographical error). In the **statistical analysis** subsection, it is unclear which variables or measurements were analyzed; these should be explicitly stated to ensure reproducibility and interpretability of the results

 Discussion 

 In the Discussion, it would be valuable to address studies that have raised precautions regarding the cytotoxic effects of essential oils, as several reports have documented that, depending on the type and concentration, certain essential oils may exhibit cytotoxic or pro-oxidant effects on mammalian cells. This aspect should be acknowledged and contextualized by incorporating the following references, among others:

 Ahn C, Lee JH, Park MJ, Kim JW, Yang J, Yoo YM, Jeung EB. Cytostatic effects of plant essential oils on human skin and lung cells. Exp Ther Med. 2020 Mar;19(3):2008-2018. doi: 10.3892/etm.2020.8460.

 Avanci-Júnior JA, Silva MF, Santos KFDP, Yoshida NC, Santos ÉL, Guterres ZR, Gouveia JG, Bogo D. Genotoxic and cytotoxic potential of essential oils from plants of the Cerrado and southern Pantanal of Mato Grosso do Sul, Brazil. Braz J Biol. 2025 Sep 29;85:e292601. 

 Orchard A, Kamatou G, Viljoen AM, Patel N, Mawela P, van Vuuren SF. The Influence of Carrier Oils on the Antimicrobial Activity and Cytotoxicity of Essential Oils. Evid Based Complement Alternat Med. 2019 Jan 14;2019:6981305. 

 Incorporating these references would enhance the scientific rigor of the discussion and demonstrate a balanced interpretation of the antimicrobial potential and possible cytotoxic implications of essential oils in biomedical applications.

Is the work clearly and accurately presented and does it cite the current literature?

Partly

If applicable, is the statistical analysis and its interpretation appropriate?

Partly

Are all the source data underlying the results available to ensure full reproducibility?

Yes

Is the study design appropriate and is the work technically sound?

Yes

Are the conclusions drawn adequately supported by the results?

Yes

Are sufficient details of methods and analysis provided to allow replication by others?

Partly

Reviewer Expertise:

Biomaterials, Bioestatistics, Microbiololgy

I confirm that I have read this submission and believe that I have an appropriate level of expertise to confirm that it is of an acceptable scientific standard, however I have significant reservations, as outlined above.
